# Epigenetic Alteration in Colorectal Cancer: A Biomarker for Diagnostic and Therapeutic Application

**DOI:** 10.1055/s-0042-1757404

**Published:** 2022-09-29

**Authors:** Hafsa Yousif Solayman Essa, Gunay Kusaf, Ozel Yuruker, Rasime Kalkan

**Affiliations:** 1 Department of Medical Genetics, Faculty of Medicine, Near East University, Nicosia, Cyprus; 2 Department of Medical Biology, Faculty of Medicine, Kyrenia University, Kyrenia, Cyprus; 3 Department of Medical Genetics, Faculty of Medicine, Cyprus Health and Social Sciences University, Guzelyurt, Cyprus

**Keywords:** colorectal cancer, DNA methylation, epigenetics

## Abstract

Colorectal cancer (CRC) is the leading cause of cancer death worldwide. A crucial process that initiates and progresses CRC is various epigenetic and genetic changes occurring in colon epithelial cells. Recently, huge progress has been made to understand cancer epigenetics, especially regarding DNA methylation changes, histone modifications, dysregulation of miRNAs and noncoding RNAs. In the “epigenome” of colon cancer, abnormal methylation of genes that cause gene alterations or expression of miRNA has been reported in nearly all CRC; these findings can be encountered in the average CRC methylome. Epigenetic changes, known as driving events, are assumed to play a dominant part in CRC. Furthermore, as epigenetic changes in CRC become properly understood, these changes are being established as clinical biomarkers for therapeutic and diagnostic purposes. Progression in this area indicates that epigenetic changes will often be utilized in the future to prevent and treat CRC.

## Introduction

In Western countries, colorectal cancer (CRC) is defined as the second most common reason of cancer deaths and has seen a marked increase in Eastern countries over the past few decades. There are three CRC types, which include sporadic, familial, and hereditary. 1 In 25% of cases there is a family history of the disease; yet, most CRCs happen randomly without such a precedent. There are four types of sporadic CRC: methylator phenotype of CpG island, hypermutated, raised microsatellite alterations at tetranucleotide repeats with metastatic behavior, and nonhypermutated. 1 In addition, familial adenomatous polyposis and hereditary nonpolyposis CRC are two hereditary types of CRC that happen because of an inherited mutation in an autosomal dominant manner. 2 It has become apparent at the occurrence of the multiphase process of CRC carcinogenesis happens due to reason of changes in epigenetics and genetic manner progressively accumulating, causing homeostatic dysregulation, leading to neoplastic transformations. 3

The role of epigenetic changes in cancer development has only been recently recognized. In 1942, Conrad H. Waddington used the concept of “epigenetics.” It involves heritable and potentially reversible changes to the genome's phenotypic expression, which alters gene expression but does not affect the deoxyribonucleic acid (DNA) primary sequence. Research has tended to focus on epigenetic modifications which contain noncoding ribonucleic acid (ncRNA), histone modifications, and DNA methylation alterations that play a part in CRC pathogenesis. 4 Although screening strategies have improved and more effective treatments are available for CRC, the prognosis of CRC remains poor. In addition, molecularly targeted agents have been only reported as active in the case of CRC in metastasis which leads the economic burden of treatment to be exponentially enhanced. Therefore, identifying robust biomarkers to diagnose and predict early-stage CRC cases is necessary. 5 Because, unlike genetic modifications, epigenetic alterations can be reversible. They are a potential new target by which in the development of prevention strategies for cancer. 2 6

### Hypomethylation in CRC

The hypomethylation of DNA significantly play a role in the development of CRC via activating the transposable elements, removing imprinting, and disrupting the stability of chromosomes. Global hypomethylation often takes place in repetitive transposable elements. 7 Aberrant LINE-1 hypomethylation is known to be linked to worse patient survival. 4 An assumption is that inadvertently activating potential proto-oncogenes could induce the methylation of SINE and LINE sequences to be abnormally hypomethylated, and in the development of CRC hypomethylated LINE-1 functionally acts. In the promoter region of CGIs (CpG islands), gene-specific hypomethylation happens in CGIs and causes oncogene to be abnormally overexpressed. 8

### Hypermethylation in CRC

Advancements in the understanding of CRC molecular pathogenesis, microsatellite instability (MSI), and chromosomal instability have been shown as primary pathways of molecular genomic instability, which mainly induces neoplasms. Recent studies have identified CpG island methylator phenotype (CIMP) as a vital process that contributes to the development of CRC. In 1999, the phenotype of CIMP was first found in colorectal tumors. 9 The CIMP is affected by numerous genes which play a crucial function within cells. 3 The hypermethylation of *CDKN2A* and *MLH1* genes' promoter region has been reported in most of the CIMP of CRC. Distinct pathological and molecular properties are found in CIMP-positive tumors, including a tendency for female gender, mucinous and poor histology, proximal location in the colon, and frequent mutations (KRAS and BRAF). 10 Suppression of DNA repair genes and tumor suppressor genes including *MGMT SEMA3F* , *APC2* , *SLC5A8* , *MLH1* , *ITGA4* , *CDKN2A P16* , *SFRP2* , *HLTF* , and *PTCH1* act significantly during progression adenomas to CRC. 11

### Epigenetic Biomarkers for CRC

The National Institutes of Health Biomarkers Definition Working Group described the expression of either “biomarker” or “biological marker” as a characteristic that is objectively measured and evaluated as an indicator of normal biological and pathological processes in 1998. According to current clinical standards, CRC biomarkers are materials by which people affected with cancer can be identified in an easy and cheap way; and the prognosis of affected people can be identified without depending on traditional classifications. 12

### DNA Methylation as Biomarker for Diagnosis

Despite improvements in studies of cancer therapy strategies, premature detection and removing of precancer lesions remains most successful in CRC and reducing cancer-related death. Nevertheless, expense, poor patient compliance, and invasiveness place a limitation on current screening methods, resulting in late diagnosis and therefore unfavorable prognosis. DNA methylation seems to occur early in tumorigenesis and is therefore indicated as a possible next generation of cancer diagnostic biomarkers. 13 14 The markers of DNA methylation in particular biofluids such as stool and blood (serum or plasma) have minimum invasiveness properties of diagnosis implemented for the screening of CRC 4 12 .

#### Diagnostic Biomarkers Based in the Blood and Stools

In the 1970s, free DNA biomarkers were discovered by studying abnormal elevations in serum DNA concentrations among cancer patients. 15 Subsequently, there has been an increased effort to expand into developing diagnostic tests based on circulating methylation DNA in the blood. There are many genes that can be utilized as blood-based methylation markers to be able to detect CRC such as *RUNX3* , *APC* , *SEPT9* , *MGMT* , *WIF1* , *hMLH1* , *RASSF2A* , *HLTF* , *VIM* , *ALX4* , *SFRP2* , *NGFR* , *NEUROG1* , and *TMEFF2* , while their range of sensitiveness and specificities are respectively 34 to 90% and 69 to 100%. 12 The methylated *SEPT9* gene is recently identified as a strong potential blood-based methylation biomarker in the case of distinguishing which samples are cancerous or not via high rate of specificity and sensitivity (respectively 90 and 72%) compared with other markers. Thus, the blood-based assay for methylated *SEPT9* is marketed by Epi proColon 1.0 (Epigenomics, Seattle, Washington, United States), ColoVantage (Quest Diagnostics, Madison, New Jersey, United States), and RealTime mS9 (Abbott Laboratories; Des Plaines, Illinois, United States) and is now marketed in several countries. 16 The GTP-binding protein is synthesized by SEPT9, and it acts significantly in the organization of cytoskeletal, division of cells, and remodeling of the membrane. In addition, many stool-based methylation biomarkers are known to detect CRC earlier, including hypermethylated HLTF, WIF1, *APC* , *VIM* , *BMP3* , *TFPI2* , *ATM* , *RASSF2A* , *SFRP2* , *NDRG4* , *CDKN2A* , *MGMT* , *GATA4* , *MLH1* , and *GSTP1* genes, which were reviewed by several publications. 12 16

The stool-DNA screening is one of the first tests accessible in trading and clinically confirmed to be used for the analysis of hypermethylated vimentin gene to be able to detect CRC (ColoSureÔ, Laboratory Corp, Burlington, North Carolina, United States). The protein of intermediate filament which is crucial in the stabilization of the cytoskeleton and is highly methylated in CRC is produced by the gene of vimentin ( *VIM* ). Respectively, high specificity and sensitivity (90 and 46%) are obtained by this detection test compared with specificities (95%) and sensitivities (14%) of fecal occult blood test. 16 The significance and credibility of methylation of DNA in the case of diagnostic biomarker are approved by scientists, extensive research has recently focused on this epigenetic alteration in different malignancies, and CRC may be detected earlier using new methylated genes. For instance, the methylation status of the gene of *ADAMTS19* is evaluated on 252 CRC-affected people by Alonso t al; it is concluded as *ADAMTS19* is epigenetically silenced in all CRC affected participants due to reason of hypermethylation. It is added by Alonso et al, that the methylated gene of *ADAMTS19* could be used as a new marker for earlier detection of CRC. 17 More research is needed to confirm these genes as diagnostic biomarkers for CRC in population-based screening.

### Methylated DNA as a Treatment Response Predictive Biomarker

It has recently been reported that several aberrantly methylated genes may act as predictive biomarkers for people with CRC. Most research has not extended beyond the phase I/II discovery phase. As a predictive marker CIMP has undergone extensive research. In 2003, people who are affected by CRC and are CIMP-positive gained benefit from the chemotherapy application of 5-flurouracil (5-FU) without the dependency of survival and mutation conditions of either TP53 or MSI, which was concluded by Van Rijnsoever et al. 18 These findings showed that people affected by CRC and in stages II and III with CIMP-positive tumors benefited from the survival rate after the treatment with 5-FU were subsequently validated. 19 However, these results were subject to challenge in additional research that studied a large patient cohort, with the patients with CIMP-positive tumors that did not receive 5-FU treatment surviving longer in comparison to patients with CIMP-negative CRCs. In the case of comparison between the CIMP-positive CRC patients and patients who have CIMP-negative tumors, shorter disease-free survival after the treatment of 5-FU in positive patients has been detected. 20 Even though the differences between the findings may have happened due to reason of the variety of inheritance or criteria of CIMP between the participants, the utilization of CIMP as the prognostic marker is promisingly indicated; and for achieving more information about the interplay between the condition of CIMP and therapeutical reactions more research is needed to be applied. The conclusions of the credible analysis about the utilization of genes that are methylated as prognostic markers will give the direction for more explorations to be applied about the reactions of methylated genes in the case of prognosis of treatment of CRC. 12

### Histone Modification: A Potential Class of Biomarkers for Colorectal Cancer

The expression of genes and condensation of DNA is controlled by chromatin, while the nucleosomal core histones are the primary protein constitutive of chromatin. The nucleosomes consist of an octamer (two copies of each histone) of four core histones H2A, H2B, H3, and H4 which are wrapped around the DNA. The histone modification happens in the tails of histones, and histone tails are acetylated, methylated, ubiquitinated, phosphorylated, and sumoylated. The nucleosomal structure is changed by controlling of transcription of associated genes and modification of posttranscription, which leads to chromatin to be either in the form of open and active called euchromatin or closed and inactive named heterochromatin. 21 For example, acetylation of H3 and H4 and also di- and trimethylation of H3 lysine 4 and H3 lysine 36 (H3K4me2, H3K4me3, H3K36me2, and H3K36me3) are enriched in euchromatin and result in transcriptional activation. Contrastingly, the transcriptionally inactive condition is determined via trimethylation of H3 lysine 27 and 9 (H3K27me3 and H3K9me3) in heterochromatin. 22 After dysregulation of histone modifications in CRC was first discovered, 23 further research revealed that this dysregulation can potentially alter gene expression patterns in CRC.

The histone modification in the early state of cancers cannot be easily distinguished due to the technic limitations of analysis in the determination of posttranslational histone modifications. Therefore, using histone modifications as disease markers is a subject of conflict. Global modifications of particular histones in the early stage of tissues have been examined for the generation of disease markers in CRC. According to research about the H3K9me2, H3K4me2, and H3K9ac modifications detection by immunohistochemical staining in metastases of the liver, it has been concluded that a high expression rate of H3K4me2 leads to opposite correlativity withal poor prognosis to be figured out. 24 In addition, other research into histone modifications in circulating nucleosomes have shown that there are reduced levels of H3K9me3 and H4K20me3 as possible CRC diagnostic biomarkers. 25 However, all of these analyses that are studied until now are phase I disease marker analyses which means they should be under the consideration of “proof of principle.” Therefore, more research is needed to be applied to determine if these alterations will be utilized as disease markers in the diagnosis or prognosis of CRC.

#### Overview of Micro-RNAs

In 1993, micro-RNAs (miRNAs) were identified in the *Caenorhabditis elegans* as negative posttranscriptional regulators. They are endogenous single-stranded small RNAs while their length is 18 to 25 nucleotides. 26 The miRNAs bind their target messenger RNAs (mRNAs) and cause target mRNA to be degraded or the process of translation of mRNA to the protein is inhibited. 27

### miRNAs in Colorectal Carcinogenesis

There are more than 800 articles on alteration in miRNA expression in CRC which has been published in the database of PubMed. 28 The tumor suppressor genes can be targeted and downregulated by overexpressed miRNAs in CRC. Furthermore, the proto-oncogenes' activity in the healthy tissues can be downregulated if miRNAs were silenced in CRC. 29 The CRCs are the process that comes from the adenocarcinoma pathway; Aslam et al concluded as the upregulated miR135a/b is related to a withal decreased level of *APC* which causes Wnt signaling pathway to be activated, and normal colonic epithelium to be transformed into adenoma. Deregulated expression of let-7 miRNA family, miR-18a and miR-143 (which all have involvement in the regulation of K-RAS expression), of miR-126 and miR-21 that regulate the phosphatidylinositol-3-kinase (PI-3-K) pathway, and a cluster of miRNAs (miR-92a, miR-17, miR-19a, miR-18a, miR-19b, and miR-20a) are linked to regulation of c-myc, which is suspected to have a role in transitioning from early adenoma to advanced adenoma. Additionally, loss of p53 function is associated with low expression levels of miR-34a family (a downstream target of p53), which indicates the role of this miRNA family in transforming adenoma to carcinoma. 30 However, the information about the miRNAs in nontraditional CRC pathways such as the de novo and serrated pathways is restricted.

### MicroRNAs as Clinically Useful Biomarkers for Colorectal Cancer

The miRNA markers can be easily extracted from various clinical sample body tissues (urine, blood, stool, saliva, etc.) and formalin-fixed paraffin-embedded tissues; also, they can be stabilized by different laboratory conditions, therefore it has attracted the interest of scientific researchers for being used as a disease marker in analyses for more than 5 years. 12

#### Use of miRNAs as Diagnostic Biomarkers in CRC

The overexpression of miR-17–3p and miR-92a were identified in CRC. 16 The high expression and secretion of miR-21 in CRC are characterized by the oncogenic activity of miRNA which makes the miR-21 to be a promising diagnostic marker of CRC in the early stage of adenoma-carcinoma. 31 The utilization of miR-21 as diagnostic marker of CRC shows high expression and secretion of miR-21 in the cells of stool or serum samples. 32 Various miRNAs (e.g., miR-155, miR-29a, miR-106a, miR-221, miR-200c, miR-135b, miR-18, let-7 g, miR-195, etc.) have been defined as diagnostic markers by Okugawa et al. 12 In addition, recent studies have been performed to identify new potency of miRNA as diagnosis markers (the abnormal expression of miRNAs in the serum, plasma, stool, or tissues of people who are affected by CRC as new disease marker candidates have been discovered. Basati et al downregulated two de novo miRNAs, miR-29b and miR-194, in participants who are affected by CRC and compared with healthy participants and concluded that miRNAs are powerful CRC serum biomarkers for early diagnosis with high sensitivity (72 and 80%) and specificity (77 and 75%). 33 According to Peng et al, 34 miR-378 and miR-145 can be used as a diagnostic marker for CRC in the early stages within high specificities (miR-378 and miR-145, 98 and 60%, respectively) and sensitivities (100%). It was shown that upregulation of an oncogenic activity of miR-17 -92 cluster containing miR-92a-2, miR-17, miR-92a-1, and miR- 20a, and downregulation of an oncosuppressive miR-143 – 145 cluster comprised of miR-143 and miR-145 in tumor tissues and suggested as potential diagnostic biomarkers for CRC.

### Use of miRNAs as a Predictive Biomarker in CRC

Accurately predicting the patient's response to a specific chemotherapeutic drug before commencing chemotherapy is extremely valuable and subject to comprehensive research. The drug resistance of miRNAs has been exposed by numerous in vitro-based analyses. For example, miR-200, miR-10b, miR-215, miR-19b, miR-192, miR-20a, miR-145, miR-21, miR-140, miR-23a, miR-34, miR-31, and miR-129 family are known to mediate 5-FU 5 resistance. 12

## Methods

This study adopted the qualitative method by inductive approaches, dependent upon the secondary sources and published resources related to epigenetic alteration in CRC.

Evidence was derived from Google Scholar, PubMed, and Science Direct.

## Discussion

Extensive study has demonstrated that the possibility of using abnormal DNA methylation and changes in ncRNAs as diagnosis marker for CRC. Further research into this promising class of biomarkers can lead to high-performance assays for preventing and managing CRC patients. The pathogenesis of CRC is directed by epigenetic processes, it cooperated with genetic mutations in the transformation of normal colon mucosa into colon cancer. A great deal of research has gone into developing 6 new epigenetic biomarker assays for noninvasive CRC diagnosis and treatment response prediction. Thus, abnormal DNA methylation and miRNA expression dysregulation could potentially be biomarkers for CRC. Progression in aberrant epigenetic modifications could lead to the exploration of robust and reliable biomarkers to improve CRC patient management.
